# Development of molecular markers based on the promoter difference of *LcFT1* to discriminate easy- and difficult-flowering litchi germplasm resources and its application in crossbreeding

**DOI:** 10.1186/s12870-021-03309-7

**Published:** 2021-11-16

**Authors:** Feng Ding, Haoran Li, Jinying Wang, Hongxiang Peng, Houbin Chen, Fuchu Hu, Biao Lai, Yongzan Wei, Wuqiang Ma, Hongli Li, Xinhua He, Shuwei Zhang

**Affiliations:** 1 Guangxi Crop Genetic Improvement and Biotechnology Key Laboratory, Guangxi Academy of Agricultural Sciences, Nanning, 530007 Guangxi China; 2 Horticultural Research Institute, Guangxi Academy of Agricultural Sciences, Nanning, 530007 Guangxi China; 3 College of Agriculture, State Key Laboratory for Conservation and Utilization of Subtropical Agro-Bioresources, Guangxi University, Nanning, 530004 Guangxi China; 4 Horticulture College, South China Agricultural University, Guangzhou, 510642 Guangdong China; 5 Institute of Tropical Fruit Trees, Hainan Academy of Agricultural Sciences/Hainan Provincial Key Laboratory of Tropical Fruit Tree Biology, Haikou, 510642 Hainan China; 6 School of Advanced Agriculture and Bioengineering, Yangtze Normal University, Chongqing, 408100 China; 7 Institute of Tropical Bioscience and Biotechnology, Chinese Academy of Tropical Agricultural Sciences, Haikou, 571101 Hainan China; 8 College of Horticulture, Hainan University, Haikou, 570228 Hainan China

**Keywords:** *Litchi chinensis*, *LcFT1*, Promoter difference, Molecular markers, Crossbreeding

## Abstract

**Background:**

Litchi is a well-known subtropical fruit crop. However, irregular bearing attributed to unstable flowering is a major ongoing problem for the development of the litchi industry. In a previous study, our laboratory proved that litchi flowering was induced by low temperature and that a *FLOWERING LOCUS T* (*FT*) homologue gene named *LcFT1* played a pivotal role in this process. The present study aimed to understand the natural variation in *FT* among litchi germplasm resources and designed markers to verify easy- and difficult-flowering litchi germplasms. A grafting experiment was also carried out to explore whether it could shorten the seedling stage of litchi seedlings.

**Results:**

Two types of *LcFT1* promoter existed in different litchi germplasm resources, and we named them the ‘easy-flowering type of *LcFT1* promoter’ and ‘difficult-flowering type of *LcFT1* promoter’, which resulted in three different *LcFT1* genotypes of litchi germplasm resources, including the homozygous easy-flowering type of the *LcFT1* genotype, homozygous difficult-flowering type of the *LcFT1* genotype and heterozygous *LcFT1* genotype of litchi germplasm resources. The homozygous easy-flowering type of the *LcFT1* genotype and heterozygous *LcFT1* genotype of the litchi germplasm resources completed their floral induction more easily than the homozygous difficult-flowering type of the *LcFT1* genotype of litchi germplasm resources. Herein, we designed two kinds of efficient molecular markers based on the difference in *LcFT1* promoter sequences and applied them to identify of the easy- and difficult-flowering litchi germplasm resources. These two kinds of molecular markers were capable of clearly distinguishing the easy- from difficult-flowering litchi germplasm resources at the seedling stage and provided the same results. Meanwhile, grafting the scion of seedlings to the annual branches of adult litchi trees could significantly shorten the seedling stage.

**Conclusions:**

Understanding the flowering characteristics of litchi germplasm resources is essential for easy-flowering litchi breeding. In the present study, molecular markers provide a rapid and accurate approach for identifying the flowering characteristics. The application of these molecular markers not only significantly shortened the artificial crossbreeding cycle of easy-flowering litchi cultivars but also greatly saved manpower, material resources and land.

**Supplementary Information:**

The online version contains supplementary material available at 10.1186/s12870-021-03309-7.

## Background

Litchi (*Litchi chinensis* Sonn.), a member of the *Sapindaceae* family, is a well-known subtropical evergreen fruit crop. Litchi has been widely cultivated in tropical and subtropical areas in the world, particularly in South China [15]. Previous production experiences have shown that a few litchi cultivars, such as ‘Sanyuehong’ and ‘Feizixiao’, need a shorter period of low temperature to complete their floral induction in October or November, and generally flower in January or February. However, most litchi cultivars must experience a longer period of low temperature in winter to complete their floral induction and then generally flower in April, approximately 2 months later than the former in South China. Thus, it is easier for the former to complete floral induction than for the latter. Therefore, they are named easy- and difficult-flowering litchi cultivars in this study. Previous production experiences have also suggested that the lack of low temperature is the main cause of unstable flowering of difficult-flowering litchi cultivars, such as ‘Nuomici’ and ‘Guiwei’, which are very popularly favoured by customers due to the high quality of their fruits.

Irregular bearing attributed to unstable flowering is a major ongoing problem for the development of the litchi industry. The unreasonable cultivation structure of easy- and difficult-flowering litchi cultivars is the main reason for the problems mentioned above. The easy-flowering litchi cultivars more easily complete floral induction than the difficult-flowering cultivars. However, few easy-flowering litchi cultivars with high quality are currently available for commercial cultivation in litchi production. Thus, it is particularly important to breed high-quality, easy-flowering litchi cultivars to develop new easy-flowering cultivars with the potential for both high quality and high yield. At the same time, there is a need to better understand the genetic and molecular mechanisms underlying the natural variation in flowering between the easy- and difficult-flowering litchi cultivars for the future work of litchi breeding.

At present, artificial crossbreeding of easy-flowering litchi cultivars has become a feasible method and can be applied to develop new easy-flowering cultivars with high quality. Marker-assisted selection (MAS) is a valuable tool to identify plants with traits of interest at an early stage in the breeding process [18]. MAS has been widely used in crop breeding [6, 8, 13, 16]. In a previous study, we demonstrated that litchi flowering was induced by low temperature and a *FLOWERING LOCUS T* (*FT*) homologue gene named *LcFT1,* which plays a pivotal role in the litchi floral transition [11]. Floral transition is one of the major phases in the life cycle of flowering plants. Reproductive success in plants relies on the proper timing of the floral transition [20]. In the model plant species *Arabidopsis*, this floral transition is coordinately regulated by four major pathways, including autonomous pathways, gibberellin pathways, photoperiod pathways and vernalization pathways [3, 5, 14, 22], which are integrated into flowering genetic pathways via a small group of floral integrators, including *FT*, *SUPPRESSOR OF OVEREXPRESSION OF CONSTANS 1* (*SOC1*) and *LEAFY* (*LFY*) [9, 20]. FT, a small and mobile protein, interacts with the bZIP transcription factor *FLOWERING LOCUS D* (*FD*) to activate the transcriptional factors *APETALA1* (*AP1*), *SOC1*, *FRUITFUL* (*FUL*) and *SEPALLATA 3* (*SEP3*), finally initiating the switch from vegetative growth to reproductive development [1, 2, 10, 23, 24, 26]. Herein, we found that two types of *LcFT1* promoters existed among different litchi germplasm resources, namely, the ‘easy-flowering type of *LcFT1* promoter’ and the ‘difficult-flowering type of *LcFT1* promoter’. We also found that the litchi germplasm resources including the easy-flowering type of the *LcFT1* promoter, were easy-flowering germplasm resources. In the present study, we designed and employed an efficient marker system based on the difference in *LcFT1* promoter sequences and applied this system to discriminate easy- from difficult-flowering litchi germplasm resources. Meanwhile, we carried out a litchi crossbreeding experiment for the breeding of easy-flowering cultivars with high quality.

## Results

### Cloning of the promoter sequences of the *LcFT1* gene and sequence analysis

**Fig. 1:**
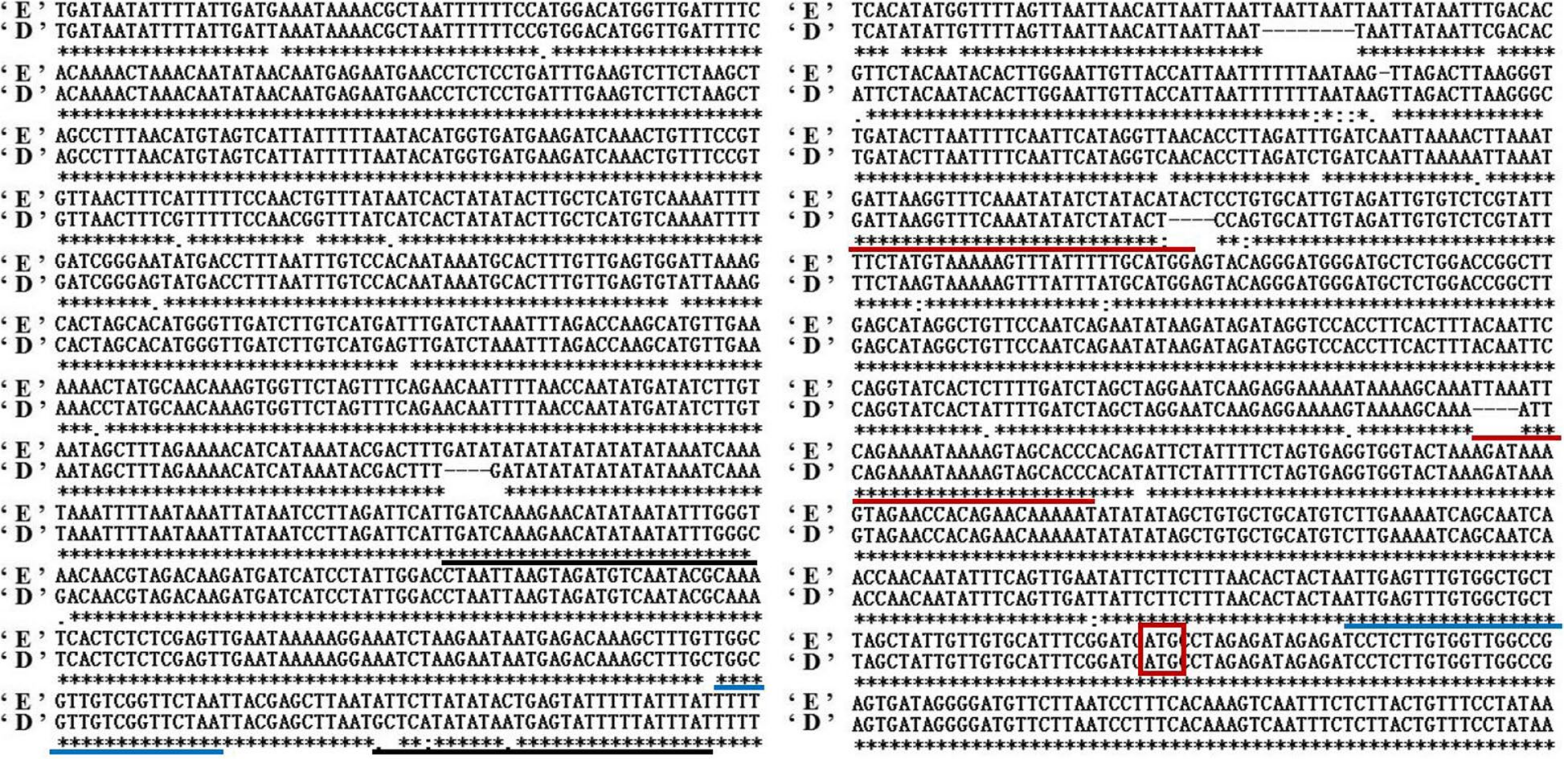
Alignment of the easy- and difficult-flowering type of *LcFT1* promoter sequences. (‘E’: easy-flowering type of the *LcFT1* promoter, ‘D’: difficult-flowering type of the *LcFT1* promoter, red underline: the location of E*LcFT1*_F and E*LcFT1*_R, black underline: the location of D*LcFT1*_F and D*LcFT1*_R, blue underline: the location of CE*LcFT1*_F and CE*LcFT1*_R, red box: start codon)

In this study, 88 litchi germplasm resources (Table S1) were used for further research and observation. We extracted their total DNA samples whose quality and quantity had met the requirements of the follow-up experiments (Fig. S1). Then, an approximately 1500-bp promoter sequence of the *LcFT1* gene was cloned from 88 litchi germplasm resources. The results showed that there were only two types of *LcFT1* promoters existing in different litchi germplasm resources named ‘easy-flowering type of *LcFT1* promoter’ and ‘difficult-flowering type of *LcFT1* promoter’, respectively. Sequence analysis showed that there were four INDELs and 36 single-nucleotide polymorphism (SNPs) between the easy- and difficult-flowering types of *LcFT1* promoter sequences (Fig. 1). The 88 litchi germplasm resources included three different *LcFT1* genotypes, namely homozygous easy-flowering type of *LcFT1* promoter litchi genotypes (NO:1–10), homozygous difficult-flowering type of *LcFT1* promoter litchi genotypes (NO:11–62), and heterozygous *LcFT1* promoter litchi genotypes (NO:63–88). They are listed in Supplemental Table 1.

### Molecular marker development

**Fig. 2:**
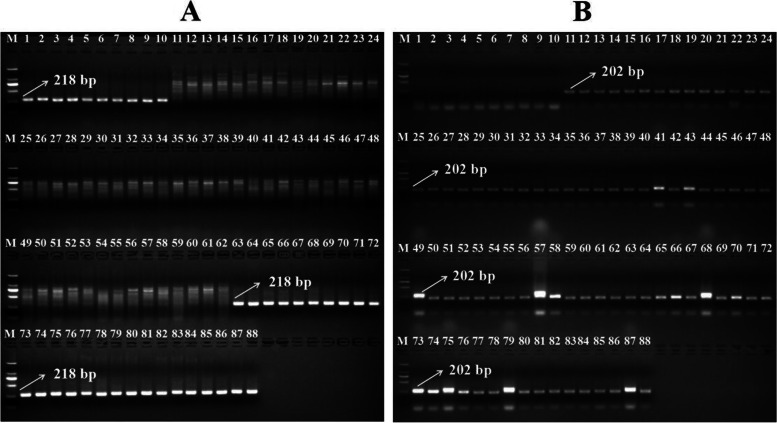
The markers E*LcFT1*_F_E*LcFT1*_R and D*LcFT1*_F_D*LcFT1*_R were verified on the 88 different litchi germplasm resources. **A** Marker E*LcFT1*_F_E*LcFT1*_R produced bands of 218, 0 and 218 bp in 10 homozygous easy-flowering type *LcFT1* promoter litchi cultivars (NO:1–10), 52 homozygous difficult-flowering type *LcFT1* promoter litchi cultivars (NO:11–62) and 26 heterozygous *LcFT1* promoter litchi cultivars (NO:63–88), respectively. **B** Marker D*LcFT1*_F_D*LcFT1*_R produced bands of 0, 202 and 202 bp in 10 homozygous easy-flowering type *LcFT1* promoter litchi cultivars (NO:1–10), 52 homozygous difficult-flowering type *LcFT1* promoter litchi cultivars (NO:11–62) and 26 heterozygous *LcFT1* promoter litchi cultivars (NO:63–88), respectively. (M: DL 2000 marker)

The experiments described above have shown that there are only two types of *LcFT1* promoters existing in different litchi germplasm resources and three different *LcFT1* genotypes. To identify the different *LcFT1* genotypes, we designed two markers according to the difference in the easy- and difficult-flowering types of *LcFT1* promoters and verified them with 88 different litchi germplasm resources. The marker E*LcFT1*_F_E*LcFT1*_R produced bands of 218, 0 and 218 bp in 10 homozygous easy-flowering type *LcFT1* promoter litchi cultivars (NO:1–10), 52 homozygous difficult-flowering type *LcFT1* promoter litchi cultivars (NO:11–62) and 26 heterozygous *LcFT1* promoter litchi cultivars (NO:63–88), respectively (Fig. 2A). The marker D*LcFT1*_F_D*LcFT1*_R produced bands of 0, 202 and 202 bp in 10 homozygous easy-flowering type of *LcFT1* promoter litchi cultivars (NO:1–10), 52 homozygous difficult-flowering type of *LcFT1* promoter litchi cultivars (NO:11–62) and 26 heterozygous *LcFT1* promoter litchi cultivars (NO:63–88), respectively (Fig. 2B). The above results showed that these two markers could clearly identify the different *LcFT1* genotypes.

**Fig. 3:**
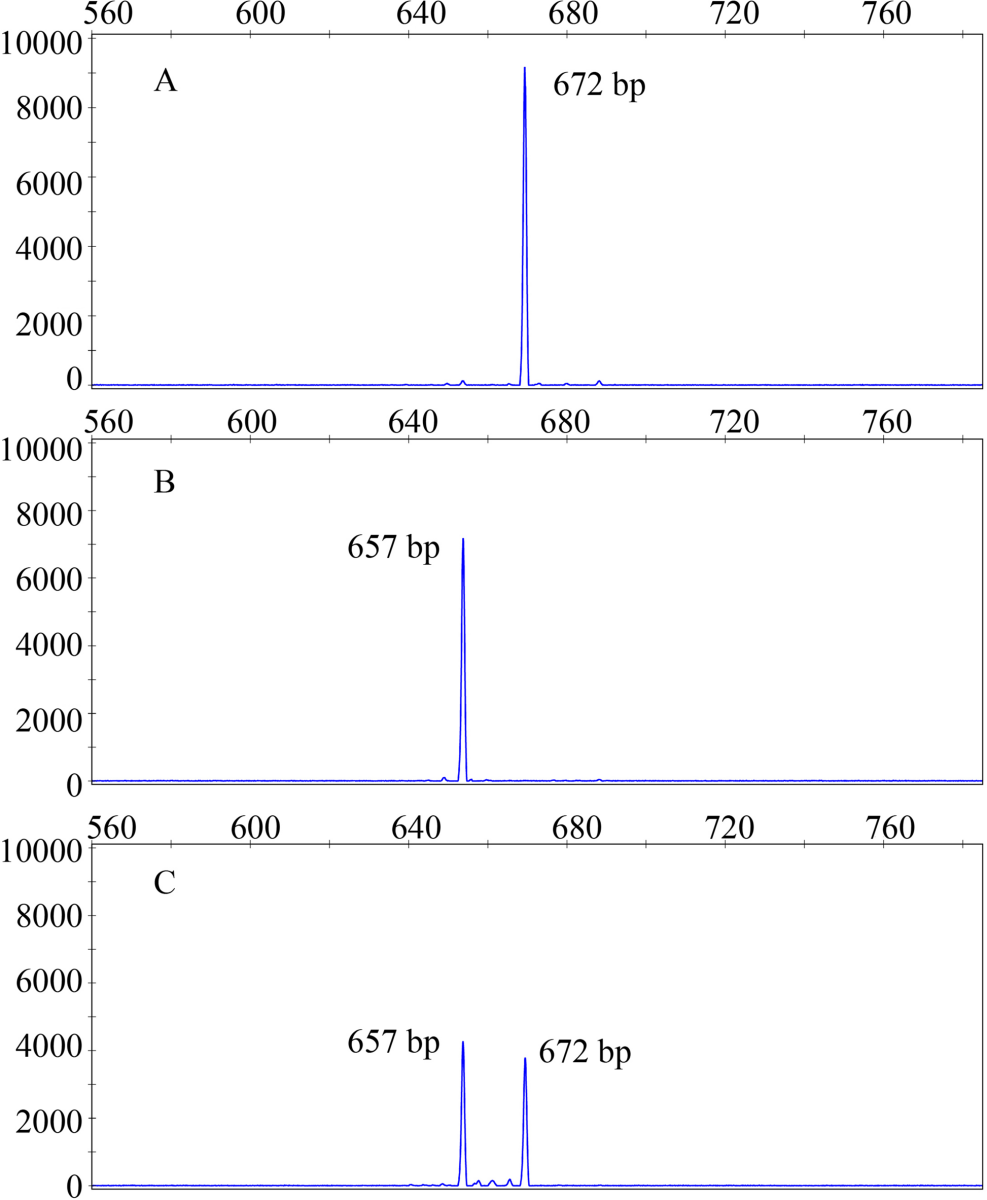
The markers CE*LcFT1*_F and CE*LcFT1*_R were verified on the 88 different litchi germplasm resources using capillary electrophoresis. The CE*LcFT1*_F and CE*LcFT1*_R markers produced bands of 672 and 657 bp for 10 homozygous easy-flowering *LcFT1* promoter litchi cultivars (NO: 1–10) and 52 homozygous difficult-flowering *LcFT1* promoter litchi cultivars (NO: 11–62), respectively (**A**, **B**), and produced 672 and 657 bp for 26 heterozygous *LcFT1* promoter litchi cultivars simultaneously (NO:62–88) (**C**). The horizontal scale indicates fragment size (in bp), and the vertical scale indicates relative fluorescence intensity (in rfu)

To further verify the accuracy and reliability of the above molecular markers, we also designed another kind of molecular marker, CE*LcFT1*_F and CE*LcFT1*_R, and used it to distinguish 88 different litchi germplasm resources. The pair of primers striding over three indels was designed in the same sequence of these two types of *LcFT1* promoters (Fig. 1). The expected product sizes were 672 bp for the easy- and 657 bp for the difficult-flowering type of the *LcFT1* promoter. Owing to the very small difference between the amplified fragments (15 bp), the method of agarose gel electrophoresis cannot easily distinguish the different *LcFT1* genotypes (Fig. S2). Therefore, the PCR products of this marker obtained from different samples were analysed by capillary electrophoresis. The results showed that the CE*LcFT1*_F and CE*LcFT1*_R markers produced 672 and 657 bp for 10 homozygous easy-flowering *LcFT1* promoter litchi cultivars and 52 homozygous difficult-flowering *LcFT1* promoter litchi cultivars, respectively (Fig. 3A, B) and produced 672 bp and 657 bp for 26 heterozygous *LcFT1* promoter litchi cultivars simultaneously (Fig. 3C). This marker could also clearly identify the different *LcFT1* genotypes and provided the same results as the above markers (E*LcFT1*_F_E*LcFT1*_R and D*LcFT1*_F_D*LcFT1*_R) and the results of promoter sequencing analysis.

### Correlation analysis between the flower formation rates and the *LcFT1* promoter types in different litchi germplasm resources

**Fig. 4:**
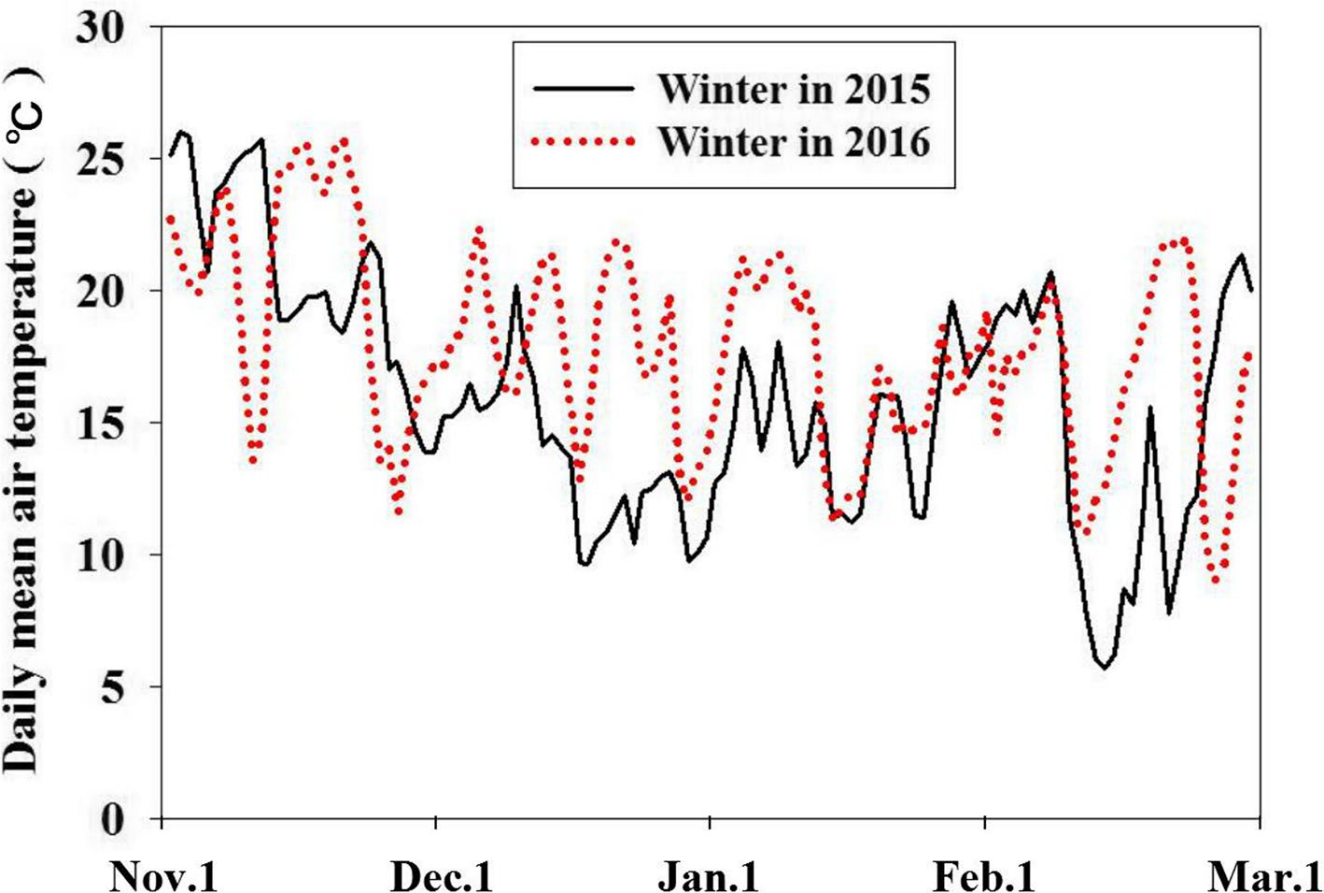
The comparison of the daily mean air temperature in winter from November 1 to March 1 between 2015 and 2016. The air temperature was obtained using an automatic temperature recording system at the experimental orchard

**Fig. 5:**
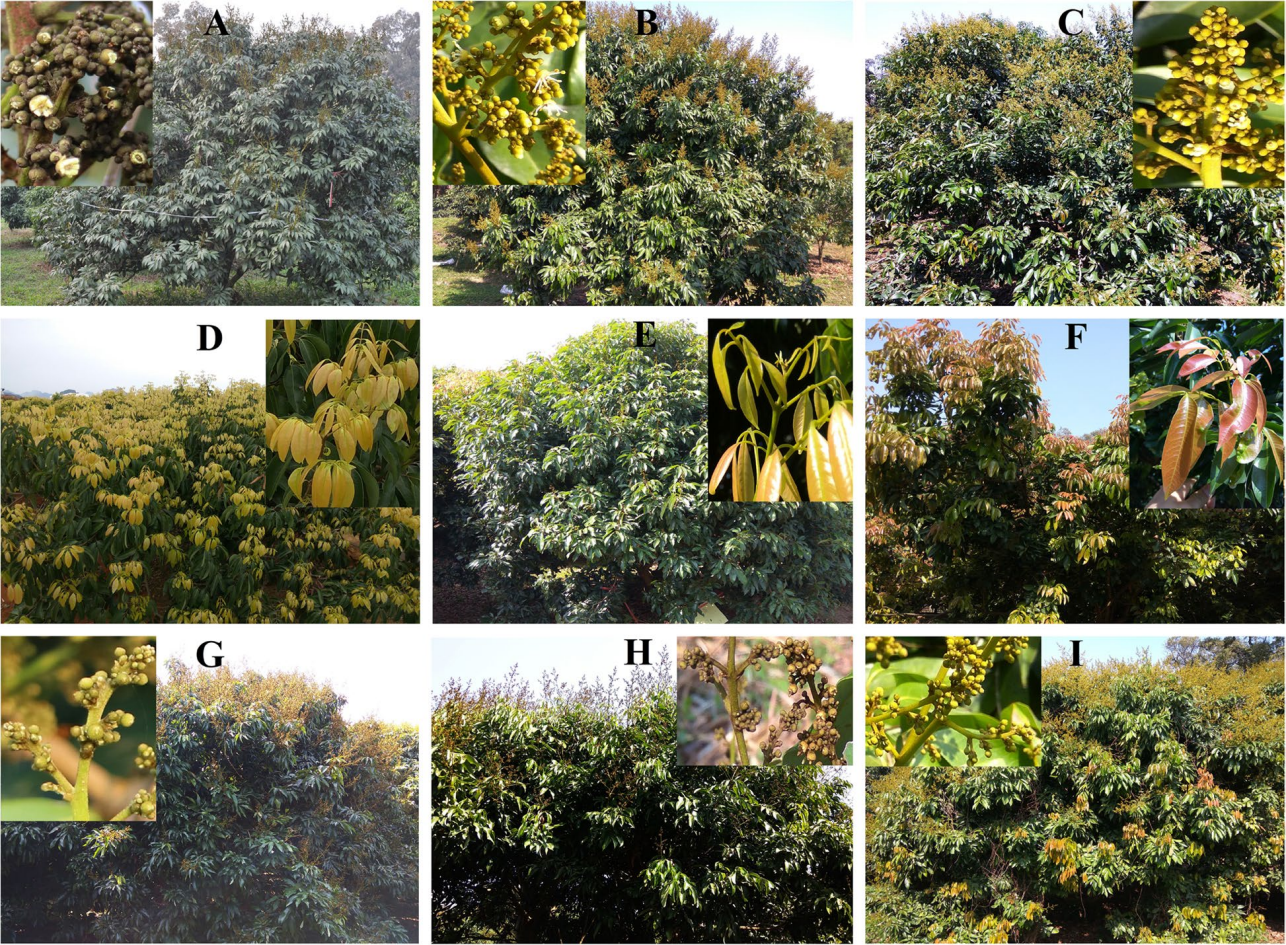
Observation of flowering in different *LcFT1* promoter types of litchi cultivars in 2017. ‘Guiwei’, ‘Nuomici’ and ‘Maguili’, three homozygous difficult-flowering type of *LcFT1* promoter litchi cultivars (**D**, **E**, **F**), could not be flowering owing to the lack of low-temperature in winter of 2016, whereas ‘Sanyuehong’, ‘Feizixiao’ and ‘Lanzhu’ (three homozygous easy-flowering type of *LcFT1* promoter litchi cultivars (**A**, **B**, **C**)) and ‘Ruanzhizaohong’, ‘Dahongpao’ and ‘Jinzhong’ (three heterozygous *LcFT1* promoter litchi cultivars, G, H, I) could be flowering well. (**A** ‘Sanyuehong’, **B** ‘Feizixiao’, **C** ‘Lanzhu’, **D** ‘Guiwei’, **E** ‘Nuomici’, **F** ‘Maguili’, **G** ‘Ruanzhizaohong’, **H** ‘Dahongpao’, and **I** ‘Jinzhong’)

The results of the above experiment showed that the 88 litchi germplasm resources included 10 homozygous easy-flowering *LcFT1* promoter litchi cultivars, 52 homozygous difficult-flowering *LcFT1* promoter litchi cultivars and 26 heterozygous *LcFT1* promoter litchi cultivars. One important discovery made in this study is that all easy-flowering litchi cultivars belong to the homozygous easy-flowering type of the *LcFT1* promoter or heterozygous *LcFT1* promoter. However, all difficult-flowering litchi cultivars belong to the homozygous difficult-flowering type of the *LcFT1* promoter. Our next aim was to better understand the correlation between flower formation rates and *LcFT1* promoter types in different litchi germplasm resources. Hence, we observed the flowering of the above 88 litchi germplasm resources and calculated their flower formation rates in 2016 and 2017. Simultaneously, we recorded the daily mean air temperatures from November 1 to March 1 in the winter of 2015 and 2016. The air temperature records showed that the winter in 2015 was much colder than the winter in 2016 (Fig. 4). The statistical results of flower formation rates showed that almost all *LcFT1* promoter types of litchi cultivars could well flower in 2016, owing to the plenty of low temperature in the winter of 2015 (Table S1). However, owing to the lack of low temperature in winter of 2016 (namely, green winter), most of the homozygous difficult-flowering type of *LcFT1* promoter litchi cultivars could not flower in 2017; even though a few cultivars did flower, the flower formation rates were generally low (Table S1). However, almost all the homozygous easy-flowering type of the *LcFT1* promoter and heterozygous *LcFT1* promoter litchi cultivars flowered (Table S1). Figure 5 shows that ‘Guiwei’, ‘Nuomici’ and ‘Maguili’, three homozygous difficult-flowering type of *LcFT1* promoter litchi cultivars, could not flower in 2017 (Fig. 5D, E, F), but ‘Sanyuehong’, ‘Feizixiao’ and ‘Lanzhu’ (three homozygous easy-flowering type of *LcFT1* promoter litchi cultivars) and ‘Ruanzhizaohong’, ‘Dahongpao’ and ‘Jinzhong’ (three heterozygous *LcFT1* promoter litchi cultivars) could be well flowering (Fig. 5A, B, C, G, H, and I). The above results indicated that those litchi germplasm resources containing the easy-flowering type of the *LcFT1* promoter can easily complete their floral induction and then flower even in the green winter.

### The application of these molecular markers in litchi crossbreeding

**Fig. 6:**
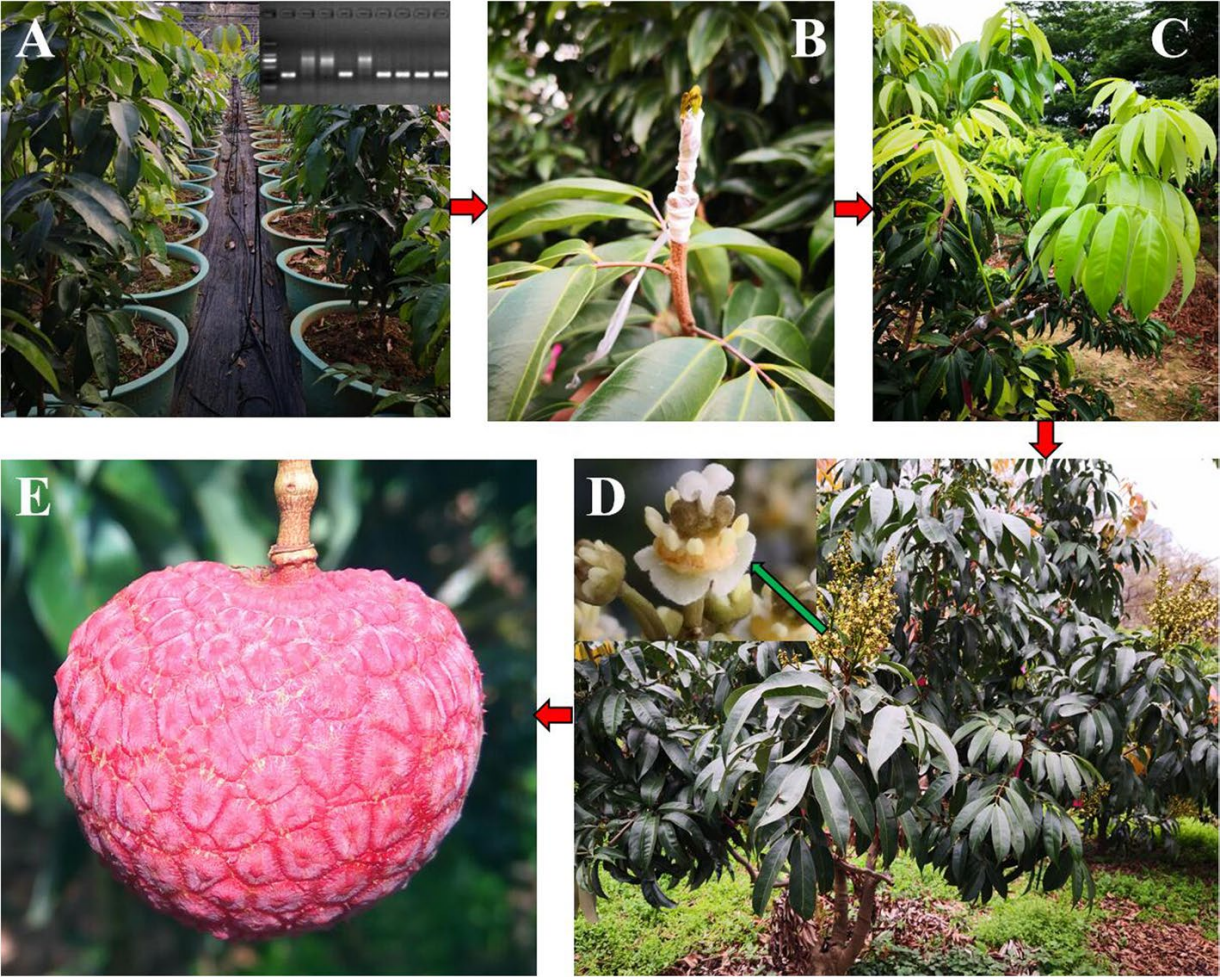
Grafting litchi seedlings to the ends of adult tree branches can significantly shorten litchi childhood. **A** The new litchi germplasms identified as true hybrids by the above developed molecular markers. **B** Annual litchi hybrid seedlings are grafted to the ends of adult tree branches. **C** Scion growth after 2 months of grafting. **D** The scion can blossom in the second year. **E** The new litchi germplasm resource ‘ZS-35’ named ‘Red Coral’ with easy flowering and good quality was created by the above methods

At present, it is particularly important to carry out the breeding of high-quality easy-flowering litchi cultivars for the healthy development of the litchi industry. The artificial crossbreeding of litchi was carried out using ‘Sanyuehong’, a homozygous easy-flowering type of *LcFT1* promoter litchi cultivar, and ‘Ziniangxi’, a homozygous difficult-flowering type of *LcFT1* promoter litchi cultivar, as male and female parents, respectively. The marker E*LcFT1*_F_E*LcFT1*_R was used to identify the seedlings. We initially obtained 387 F_1_ hybrid seedlings. However, the PCR amplification was absent in some seedlings (Fig. S3), indicating that these seedlings do not acquire the easy-flowering type of the *LcFT1* gene from the male parent of Sanyuehong, and they are false hybrid seedlings. Finally, we obtained 216 true F_1_ hybrid seedlings, from which a target band of 218 bp was amplified with the marker E*LcFT1*_F_E*LcFT1*_R (Fig. S3). Simultaneously, we found that these true heterozygous *LcFT1* promoter types of F_1_ hybrid seedlings can easily complete floral induction even in the green winter, which was consistent with our findings above. Generally, it takes approximately 5 years or longer for litchi seedlings to flower. We grafted litchi seedlings to the end cycle of adult large branches, and the scion could bloom and bear fruit in the second year (Fig. 6), which significantly shortens the litchi hybrid breeding cycle. Through the above methods, we created many litchi germplasm resources with easy flowering and good quality in a very short time, such as ‘ZS-35’ named ‘Red Coral’ (Fig. 6E).

## Discussion

Litchi is an important subtropical evergreen fruit crop with high commercial value due to its high nutritional value and favourable taste. However, unstable yield attributed to the unreasonable structure of the easy- and difficult-flowering litchi cultivars of cultivation is a major ongoing problem for litchi producers in China, with difficult-flowering cultivars accounting for the predominant proportion. Once the low temperature in winter is insufficient, most difficult-flowering litchi cultivars cannot complete their floral induction. One of the main reasons for this unreasonable structure is the lack of high-quality easy-flowering litchi cultivars. Hence, the breeding of easy-flowering litchi cultivars with high quality is particularly important for the development of new cultivars for the healthy development of the litchi industry. The traditional breeding process is tedious and time-consuming with a quite low success rate. Thus, new approaches are needed to accelerate the development of new cultivars with the potential for both high quality and high yield. The CRISPR/Cas system has been widely applied in plant breeding, which enables promising new opportunities to create genetic diversity for breeding [4, 17, 21, 27]. Unfortunately, due to the lack of a genetic transformation system for litchi, the CRISPR/Cas system cannot be used in litchi genome improvement at present, although it can greatly accelerate the breeding process. Additionally, MAS is a process in which morphological, biochemical, and/or DNA markers are utilized for the indirect selection of desirable traits that could be utilized in breeding at the seedling stage [8, 28]. MAS can significantly enhance genetic gain, especially in cases where phenotypes are highly dependent on specific environmental conditions and selection is time-consuming or where traits such as grain quality and disease resistance are pivotal [19]. The selection of an appropriate MAS is the key for the success of the selection of desirable traits.

In this study, we found that two types of *LcFT1* promoters existed in different litchi germplasm resources named ‘easy-flowering type of *LcFT1* promoter’ and ‘difficult-flowering type of *LcFT1* promoter’, which resulted in three different *LcFT1* genotypes of litchi cultivars, including homozygous easy-flowering type of *LcFT1* promoter litchi cultivar, homozygous difficult-flowering type of *LcFT1* promoter litchi cultivar and heterozygous *LcFT1* promoter litchi cultivar. Therefore, in the future, CRISPR can be used to precisely modify the difficult-flowering type of the *LcFT1* promoter. It will be possible to obtain easy-flowering cultivars with high quality more quickly. The correlation analysis between the flower formation rates and the *LcFT1* promoter types among 88 different litchi germplasm resources showed that almost all the homozygous easy-flowering type of *LcFT1* promoter litchi cultivars and heterozygous *LcFT1* promoter litchi cultivars are easy-flowering litchi cultivars, which easily complete their floral induction even in a green winter. However, almost all the homozygous difficult-flowering type of *LcFT1* promoter litchi cultivars are difficult-flowering litchi cultivars, and it is more difficultly to complete their floral induction in the green winter. This important and novel finding is crucial to the crossbreeding of litchi. However, some homozygous difficult flowering genotypes, including ‘Huaizhi’, ‘Xiapuli’, ‘Shuimi’, ‘Kaleka’, and ‘Seedling tree-11’, could flower well in green winters with higher flowering rates than some heterozygous easy flowering genotypes. Litchi flowering is a very complicated biological process that is influenced by various environmental and endogenous signals. Chilling is an irreplaceable factor for litchi flowering, and abiotic stress, such as water, nitric oxide, and reactive oxygen species, could promote flowering in litchi [7, 30]. Tree growth and nutritional status are also important factors affecting litchi flowering. The difference in these factors may be attributed to the higher flowering rates of some homozygous difficult flowering genotypes than the flowering rates of some of the heterozygous easy flowering types in the green winter.

Based on the above results, two molecular markers (E*LcFT1*_F_E*LcFT1*_R and D*LcFT1*_F_D*LcFT1*_R) were designed according to the sequence difference between the easy- and difficult-flowering types of the *LcFT1* promoter and then used to verify 88 different litchi germplasm resources by agarose gel electrophoresis. The results showed that these markers can efficiently identify easy- and difficult-flowering litchi germplasm resources (Fig. 2). To verify the accuracy and reliability of the above molecular marker, we designed another kind of molecular marker, CE*LcFT1*_F and CE*LcFT1*_R, and then used that molecular marker to distinguish the above 88 different litchi germplasm resources by capillary electrophoresis. The results showed that this marker could also efficiently identify easy- and difficult-flowering litchi germplasm resources (Fig. 3), and the same results were obtained with the above two molecular markers, which also further verified the accuracy and reliability of the molecular marker development of principle. However, the cost of capillary electrophoresis is much more expensive than the cost of agarose electrophoresis. Thus, the molecular markers E*LcFT1*_F_E*LcFT1*_R and D*LcFT1*_F_D*LcFT1*_R are the first method for the identification of easy- and difficult-flowering characteristics of litchi. Unlike annual herbs, as litchi is a subtropical perennial evergreen fruit crop, its childhood phase is very long (approximately five years or more), which makes the cycle of artificial litchi crossbreeding especially long. In addition, the litchi tree body is very large and covers a large amount of land and is not conducive to carrying out artificial crossbreeding. Thus, MAS is especially important for identifying litchi germplasm resources with traits of interest at an early stage in artificial crossbreeding. Herein, we designed and employed two efficient marker systems and found that they can not only significantly shorten the litchi crossbreeding cycl, but also greatly save manpower, material resources and land. Meanwhile, the above marker systems play an important role in the regional promotion of litchi cultivars. The cultivars identified as difficult-flowering litchi germplasm resources are suitable for cultivation in late-ripening producing areas with sufficiently low temperature in winter and overcome the disadvantages of cultivars that are difficult to flower. The cultivars identified as easy-flowering litchi germplasm resources can be planted in early-ripening producing areas where low temperatures are insufficient in winter, giving play to the advantages of easy flowering and early maturation. The identified cultivars can avoid the heavy economic loss caused by the blind promotion of litchi cultivars and have a wide range of application prospects.

Generally, the qualities of difficult-flowering litchi cultivars are much better than the qualities of easy-flowering cultivars. At present, only a few easy-flowering cultivars have been cultivated on a commercial scale in China even though they an easily complete their floral induction. However, they are not very favoured by customers due to their poor qualities, including acidic taste and rough flesh. To breed easy-flowering litchi cultivars with high quality for the optimization of the cultivation structure of litchi cultivars, we carried out a crossbreeding experiment. According to our studies, homozygous easy-flowering *LcFT1* promoter litchi germplasm resources and high-quality difficult-flowering litchi germplasm resources are the best choices for parents to breed easy-flowering litchi cultivars with high quality. In this study, we identified 10 homozygous easy-flowering types of *LcFT1* promoter litchi germplasm resources. ‘Sanyuehong’, a homozygous easy-flowering type of the *LcFT1* promoter litchi cultivar that we had already identified, was used as the male parent, and ‘Ziniangxi’, a high quality and homozygous difficult-flowering type of the *LcFT1* promoter litchi cultivar, was used as the female parent. Through screening with the molecular marker E*LcFT1*_F_E*LcFT1*_R, we finally obtained 216 pure heterozygous F_1_ hybrid seedlings, which also showed the characteristics of easy flowering even in the green winter. This experimental result further validates the reliability of these molecular markers. The 171 F1 hybrid seedlings are not produced target bands. This result indicated that they were not hybrid offspring of the male parent of Sanyuehong. Because litchi is monoecious, the 171 seedlings may be the offspring of self-pollination due to imperfectly emasculation. Besides, in generally the female and male flowers were open two or three times in batches. It may have another batch of female flowers after cross-pollination and produce unexpected fruits in case of incomplete removement of female flowers. We also found that grafting litchi seedlings to the ends of adult tree branches can significantly shorten the litchi childhood phase, which further shortens the litchi crossbreeding cycle. Our work has paved a good foundation for the future breeding of high-quality easy-flowering litchi cultivars.

In this study, we developed two types of molecular markers to discriminate easy- from difficult-flowering litchi germplasm resources based on the promoter difference of *LcFT1*, and we successfully applied them in litchi crossbreeding, particularly in the breeding of new easy-flowering cultivars with high quality. However, there are still many key issues, such as whether specific regulatory elements existing within the easy-flowering type of *LcFT1* promoter sequences are functionally active and lead to those litchi germplasm resources containing easy-flowering type of *LcFT1* promoter that can easily complete their floral induction and why grafting litchi seedlings to the ends of adult tree branches can significantly shorten litchi childhood phase requires being further elucidated. These will be the main directions of our future research. Simultaneously, the breeding of new high-quality easy-flowering cultivars will be another main direction of our future work for the optimization of the cultivation structure of litchi cultivars to make an important contribution to the healthy development of the litchi industry.

## Conclusions

The present study indicates that the natural variation in litchi *FT* determines the flowering characteristics of litchi. Molecular markers used to identify easy- and difficult-flowering litchi germplasms were developed. Moreover, grafting litchi seedlings to the ends of adult large branches could significantly shorten the seedling stage.

## Methods

### Plant materials and DNA extraction

Leaves of 88 main litchi germplasm resources were sampled in the experimental orchard of South China Agricultural University (Guangzhou, Guangdong, China). The 88 litchi germplasm resources are listed in Supplemental Table 1. The typical easy-flowering cultivars include ‘Sanyuehong’, ‘Hemaoli’, ‘Feizixiao’, ‘D11’, and ‘Lanzhu’, etc. They need a shorter period of low temperature to complete their floral induction and generally flower in January or February. The typical difficult-flowering cultivars include ‘Guiwei’, ‘Nuomici’, ‘Maguili’, ‘Yuhebao’, and ‘Ziniangxi’, They generally need a longer period of low temperature in winter to complete their floral induction and flower in April. Their total DNA samples were extracted following the protocol of Doyle and Doyle [12]. The quantity of the isolated total DNA samples was evaluated with a spectrophotometer (Eppendorf, Germany) and their quality was confirmed by electrophoresis in a 0.8% agarose gel. The isolated DNA samples were diluted to 20 ng/μL and used immediately for PCR amplification or stored at − 20 °C.

### Cloning of the promoter sequences of the *LcFT1* gene and sequence analysis

Based on the genome databases established previously by our teams, the promoter sequences of *LcFT1* were cloned from the 88 litchi germplasm resources mentioned above with the primers *LcFT1*_F1 and *LcFT1*_R1. Primer sequences are listed in Supplemental Table 2. PCR amplifications were performed in a 25 μL reaction volume containing 50 ng of template DNA, 2.5 μL of 10× PCR buffer (with MgCl_2_), 0.5 mM dNTPs, 1.0 μL of each forward and reverse primer (10 pmol/μL), and 0.5 U X5 high-fidelity DNA Polymerase (Mei5 Biotechnology Co., Ltd., China). PCR was carried out in a Veriti® thermal cycler (Applied Biosystems, Foster City, CA, USA) using the following conditions: initial denaturation at 95 °C for 2 min followed by 35 cycles of 94 °C for 25 s, 55 °C for 30 s and 68 °C for 90 s, with an extension at 68 °C for 5 min, and final cooling to 4 °C. The PCR products were analysed by 1.5% agarose gel electrophoresis to verify the size and to ensure the specific amplification, then were cloned into the M5 HiPer pTOPO-Blunt vector (Mei5 Biotechnology Co., Ltd., China) and sequenced (Sangon Biotech, Shanghai, China). Promoter sequence analysis was carried out with online software PlantPAN.

### Molecular marker primer design for distinguishing easy- from difficult-flowering litchi germplasm resources by agarose gel electrophoresis

Two molecular markers were designed and used to distinguish easy- from difficult-flowering litchi germplasm resources using agarose gel electrophoresis. One set of primers, namely, E*LcFT1*_F and E*LcFT1*_R, was designed based on the specific sequence and its flanking regions in the easy-flowering type of *LcFT1* promoter sequence (Fig. 1). The expected PCR product sizes were 218 bp for the easy- and 0 bp for the difficult-flowering type of *LcFT1* promoter. The other set of primers, namely, D*LcFT1*_F and D*LcFT1*_R, was designed based on the specific sequence and its flanking regions in the difficult-flowering type of the *LcFT1* promoter sequence (Fig. 1). The expected PCR product sizes were 0 bp for easy-flowering and 202 bp for difficult-flowering *LcFT1* promoters. The sequences of these primers are listed in Supplemental Table 2. PCR amplifications was performed in a 25-μL reaction volume containing 20 ng of template DNA, 1.0 U EX Taq DNA polymerase (TaKaRa, Japan), 2.5 μL of 10x PCR buffer (with MgCl_2_), 0.5 mM dNTPs, and 0.5 μl of each primer (10 pmol/μl). The PCR amplifications were performed by using a gradient thermal cycler (Eppendorf ep™, Germany) with initial denaturation at 94 °C for 4 min followed by 35 cycles of denaturation at 94 °C for 30 s, annealing at 58 °C for 30 s and extension at 72 °C for 30 s, with an extension at 72 °C for 5 min, and final cooling to 4 °C. PCR products obtained from different samples under identical conditions of amplification were analysed by 1.5% agarose gel electrophoresis to verify the size and to ensure the specific amplification. The approximate size was evaluated by comparison to a 2000 bp DNA marker.

### Design of another kind of molecular marker primer for distinguishing easy- from difficult-flowering litchi germplasm resources by capillary electrophoresis

Another kind of molecular marker, namely, CE*LcFT1*_F and CE*LcFT1*_R, was also designed and used to distinguish easy- from difficult-flowering litchi germplasm resources using capillary electrophoresis. There were four small insertions and deletions (INDELs) between the easy- and difficult-flowering types of the *LcFT1* promoter sequence. This pair of primers of CE*LcFT1*_F and CE*LcFT1*_R stride over three indels and were designed in the same sequence of these two types of *LcFT1* promoters (Fig. 1). The expected product sizes were 672 bp for the easy- and 657 bp for the difficult-flowering types of the *LcFT1* promoter. The sequences of these primers are listed in Supplemental Table 2. PCR amplification of this marker was performed according to the method described in section 2.2. PCR products obtained from different samples were analysed by capillary electrophoresis to verify the size and to ensure specific amplification. The amplification products were diluted 20-fold, and 1 μL of the diluted products was combined with 9.8 μL ddH_2_O and 0.20 μL of the GeneScan-500 (ROX) size standard (Applied Biosystems). Samples were denatured at 95 °C for 5 min, quickly cooled on ice, and then genotyped on an ABI PRISM® 3730XL Capillary DNA Sequencer/Genotyper using GeneScan® 3.7 and Genotyper® 3.7 software (Applied Biosystems).

### Determination of the reliability and accuracy of the above molecular markers

To determine the reliability and accuracy of the molecular markers mentioned above, 88 litchi germplasm resources were used for testing. These resources included the homozygous easy-flowering type of *LcFT1* promoter litchi cultivars, the homozygous difficult-flowering type of *LcFT1* promoter litchi cultivars and the heterozygous *LcFT1* promoter litchi cultivars. The total DNA samples of these litchi germplasm resources were used as templates, and PCR amplifications of these markers were performed using the molecular marker primers mentioned in sections 2.3 and 2.4, respectively according to the procedures of PCR amplification, agarose gel electrophoresis and capillary electrophoresis mentioned above.

The specificity and accuracy of the markers E*LcFT1*_F and E*LcFT1*_R for the presence of specific PCR products with template DNA samples isolated from the above litchi germplasm resources were tested by analysing the PCR-amplified products on 1.5% agarose gel electrophoresis. The expected product sizes were 218 bp, 0 bp and 218 bp for homozygous easy-flowering type of *LcFT1* promoter litchi cultivars, homozygous difficult-flowering type of *LcFT1* promoter litchi cultivars and heterozygous *LcFT1* promoter litchi cultivars, respectively. The specificity and accuracy of the markers D*LcFT1*_F and D*LcFT1*_R for the presence of specific PCR products with template DNA isolated from the above litchi germplasm resources were also tested by analysing the PCR-amplified products on agarose gel using electrophoresis. The expected product sizes were 0 bp, 202 bp and 202 bp for homozygous easy-flowering type of *LcFT1* promoter litchi cultivars, homozygous difficult-flowering type of *LcFT1* promoter litchi cultivars and heterozygous *LcFT1* promoter litchi cultivars, respectively.

The specificity and accuracy of the markers CE*LcFT1*_F and CE*LcFT1*_R for the presence of specific PCR products with template DNA samples isolated from the above litchi germplasm resources were tested by analysing the PCR amplified products using capillary electrophoresis. The expected product size was 672 bp for homozygous easy-flowering type *LcFT1* promoter litchi cultivars. The expected product size was 657 bp for homozygous difficult-flowering type *LcFT1* promoter litchi cultivars. The expected product sizes were 672 bp and 657 bp for heterozygous *LcFT1* promoter litchi cultivars simultaneously.

### Correlation analysis between the flower formation rates and the *LcFT1* gene promoter types in different litchi germplasm resources

Observation of flowering timings of different litchi germplasm resources: The field experiments were conducted at the experimental orchard of South China Agricultural University (Guangzhou, Guangdong, China) from 2015 to 2017. Eighty-eight litchi germplasm resources were used for this study. Three trees were chosen for each litchi cultivar, and 50 branches were chosen for each tree to calculate the flower formation rate for each cultivar (flower formation rate = flowering branches/150 × 100%). Simultaneously, we recorded the daily mean air temperatures from November 1 to March 1 in winter with high-accuracy temperature recorder. Finally, we carried out correlation analysis between the flower formation rates and the *LcFT1* gene promoter types in different litchi germplasm resources using the partial correlation analysis of SPSS18.0.

### Applications of molecular markers in litchi crossbreeding

Litchi crossbreeding experiment: The crossbreeding experiments were conducted at the experimental orchard of Guangxi Academy of Agricultural Sciences (Nanning, Guangxi, China). The cultivars of Sanyuehong and Ziniangxi were used as male and female parents. Pollen collection, dehydration, and long-term storage for litchi were performed based on the method described by Wang et al. [25]. The artificial crossbreeding of litchi cultivars was carried out following the protocol of Zhang and Liu [29]. The seedlings of the F_1_ population were grown in an experimental orchard in Xixiangtang District, Nanning. Leaves were sampled from the seedlings of the F_1_ population. All leaf samples were frozen immediately in liquid nitrogen after harvest and then stored at − 20 °C until further processing for DNA extraction. The identification of a heterozygous F_1_ population derived from the cross between the Sanyuehong and Ziniangxi was carried out with the above molecular markers (E*LcFT1*_F and E*LcFT1*_R) with the detailed methods described above. Finally, we grafted one-year-old litchi hybrid seedlings to the ends of adult tree branches and observed their flower formation rates according to the method described above. Healthy rootstocks and scion branches were selected, and the scion was moisturized before grafting. The stock is thicker than the scion. Grafting was carried out on sunny days at temperature of 20 to 30 °C from March to May. We ensured that the cutting surface of the rootstock and the scion was consistent and butt, and the cambium layers were carefully aligned. The union of rootstock and scion was wrapped with a wax band to prevent drying out.

## Supplementary Information

**Additional file 1: Figure S1.** Agarose gel electrophoresis of DNA samples extracted from 88 litchi germplasm resources (M: DL2000 marker; names of 1–88 litchi cultivars are listed in Supplemental Table 1).

**Additional file 2: Figure S2.** The markers CE*LcFT1*_F and CE*LcFT1*_R were verified on the 88 different litchi germplasm resources with agarose gel electrophoresis. The expected product sizes were 672 bp and 657 bp for the easy- and difficult-flowering types of the *LcFT1* promoter, respectively. The difference between the amplified fragments is very small (15 bp). Thus, agarose gel electrophoresis canot clearly distinguish the different *LcFT1* genotypes. (M: DL 2000 marker).

**Additional file 3: Figure S3.** Identification of the F_1_ population derived from the cross between Sanyuehong and Ziniangxi with the marker E*LcFT1*_F_E*LcFT1*_R. (M: DL 2000 marker; 1–48: part of the hybrid seedlings; Note: No target 218-bp band of seedlings is a false hybrid seedling).

**Additional file 4: Table S1.** Litchi germplasm resources used in this study.

**Additional file 5: Table S2.** Primers used in this study.

## Data Availability

All data generated or analysed during this study are included in this published article and its supplementary information files.
