# Supplementary figures and images for: Development of molecular markers based on the promoter difference of LcFT1 to discriminate easy- and difficult-flowering litchi germplasm resources and its application in crossbreeding
Source: BMC Plant Biol. 2021 Nov 16;21:539. doi: 10.1186/s12870-021-03309-7 (PMC8594225; doi:10.1186/s12870-021-03309-7)

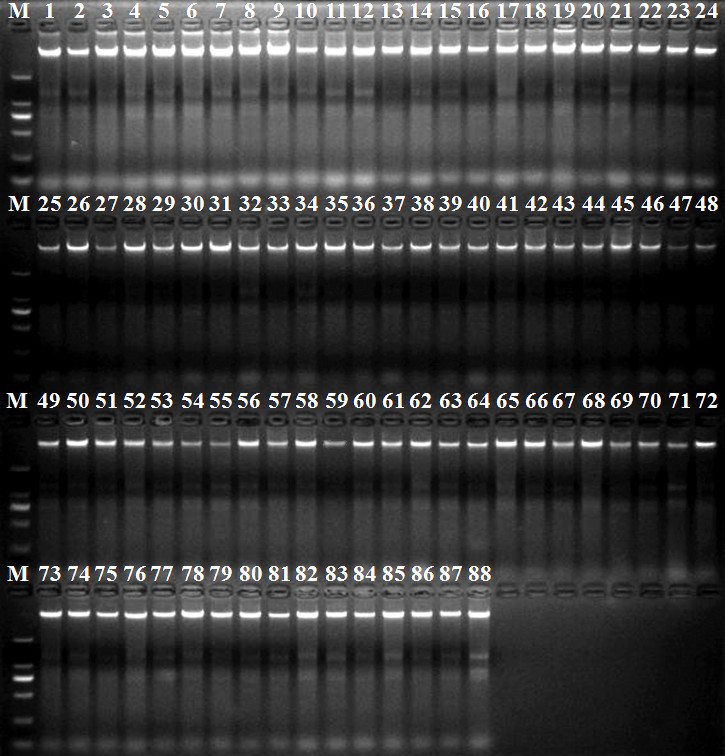

Supplement: Supplementary file 1 — Additional file 1: Figure S1. Agarose gel electrophoresis of DNA samples extracted from 88 litchi germplasm resources (M: DL2000 marker; names of 1–88 litchi cultivars are listed in Supplemental Table 1). [file 12870_2021_3309_MOESM1_ESM.jpg]

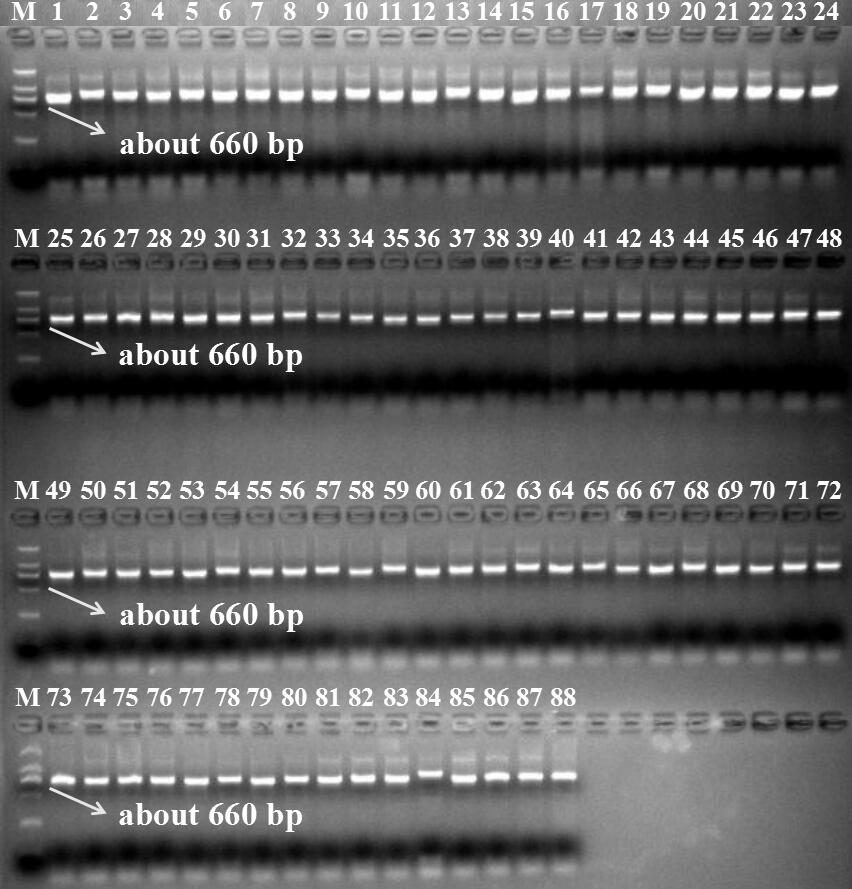

Supplement: Supplementary file 2 — Additional file 2: Figure S2. The markers CELcFT1_F and CELcFT1_R were verified on the 88 different litchi germplasm resources with agarose gel electrophoresis. The expected product sizes were 672 bp and 657 bp for the easy- and difficult-flowering types of the LcFT1 promoter, respectively. The difference between the amplified fragments is very small (15 bp). Thus, agarose gel electrophoresis canot clearly distinguish the different LcFT1 genotypes. (M: DL 2000 marker). [file 12870_2021_3309_MOESM2_ESM.jpg]

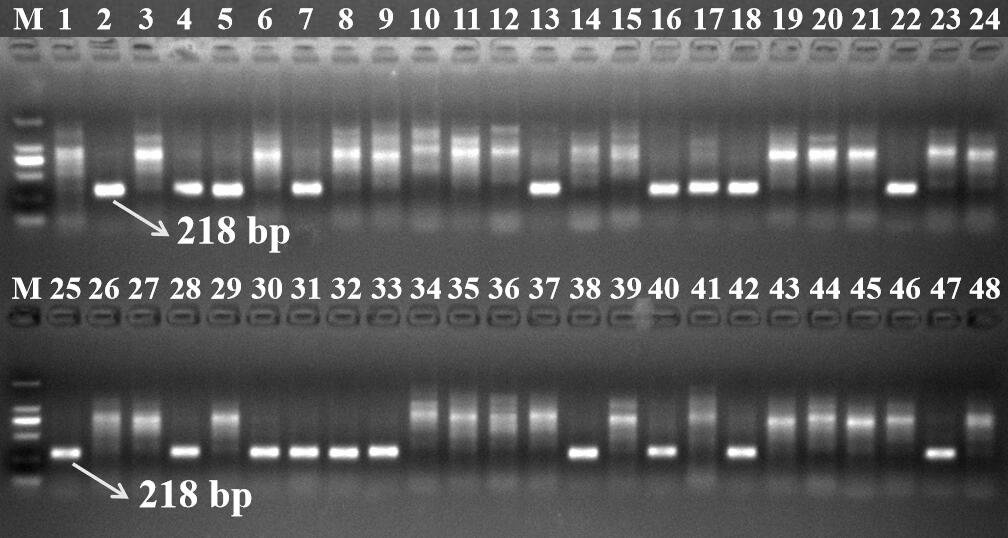

Supplement: Supplementary file 3 — Additional file 3: Figure S3. Identification of the F1 population derived from the cross between Sanyuehong and Ziniangxi with the marker ELcFT1_F_ELcFT1_R. (M: DL 2000 marker; 1–48: part of the hybrid seedlings; Note: No target 218-bp band of seedlings is a false hybrid seedling). [file 12870_2021_3309_MOESM3_ESM.jpg]
