# Supplementary material for: Development of molecular markers based on the promoter difference of LcFT1 to discriminate easy- and difficult-flowering litchi germplasm resources and its application in crossbreeding
Source: BMC Plant Biol. 2021 Nov 16;21:539. doi: 10.1186/s12870-021-03309-7 (PMC8594225; doi:10.1186/s12870-021-03309-7)
Supplement: Supplementary file 4 — Additional file 4: Table S1. Litchi germplasm resources used in this study. [file 12870_2021_3309_MOESM4_ESM.doc]

Supplemental Table 1. Litchi germplasm resources used in this study

| Cultivar NO | Cultivar names | *LcFT1* promoter types | Flower formation rates(2016) | Flower formation rates(2017) |
| --- | --- | --- | --- | --- |
| 1 | ‘Lanzhu’ | homozygous easy-flowering type | 100.00% | 100.00% |
| 2 | ‘Sanyuehong’ | homozygous easy-flowering type | 100.00% | 100.00% |
| 3 | ‘Feizixiao’ | homozygous easy-flowering type | 100.00% | 100.00% |
| 4 | ‘Hemaoli’ | homozygous easy-flowering type | 100.00% | 98.67% |
| 5 | ‘9918’ | homozygous easy-flowering type | 90.00% | 94.00% |
| 6 | ‘99112’ | homozygous easy-flowering type | 92.00% | 90.00% |
| 7 | ‘99119’ | homozygous easy-flowering type | 100.00% | 96.00% |
| 8 | ‘99149’ | homozygous easy-flowering type | 83.33% | 92.67% |
| 9 | ‘D11’ | homozygous easy-flowering type | 90.67% | 90.00% |
| 10 | ‘D13’ | homozygous easy-flowering type | 96.67% | 88.00% |
| 11 | ‘Congxing’ | homozygous difficult-flowering type | 90.67% | 0.00% |
| 12 | ‘Zaopu’ | homozygous difficult-flowering type | 94.67% | 6.67% |
| 13 | ‘Xiangli’ | homozygous difficult-flowering type | 84.67% | 0.00% |
| 14 | ‘Wanpu’ | homozygous difficult-flowering type | 76.67% | 0.00% |
| 15 | ‘Tongzai’ | homozygous difficult-flowering type | 90.67% | 10.00% |
| 16 | ‘Dongliuyihao’ | homozygous difficult-flowering type | 85.33% | 0.00% |
| 17 | ‘Nanhaili’ | homozygous difficult-flowering type | 66.00% | 0.00% |
| 18 | ‘Nuomici’ | homozygous difficult-flowering type | 92.00% | 0.00% |
| 19 | ‘Xijiaozi’ | homozygous difficult-flowering type | 89.33% | 0.00% |
| 20 | ‘Jidi’ | homozygous difficult-flowering type | 99.33% | 12.67% |
| 21 | ‘Longyanben’ | homozygous difficult-flowering type | 51.33% | 0.00% |
| 22 | ‘Hehuadahongli’ | homozygous difficult-flowering type | 86.67% | 23.33% |
| 23 | ‘TaiSo Bengal’ | homozygous difficult-flowering type | 96.00% | 16.67% |
| 24 | ‘Guiwei’ | homozygous difficult-flowering type | 90.33% | 0.00% |
| 25 | ‘Huaizhi’ | homozygous difficult-flowering type | 100.00% | 85.00% |
| 26 | ‘Maguili’ | homozygous difficult-flowering type | 98.00% | 0.00% |
| 27 | ‘Yingzhizaohong’ | homozygous difficult-flowering type | 98.67% | 0.00% |
| 28 | ‘Xiapuli’ | homozygous difficult-flowering type | 88.00% | 80.67% |
| 29 | ‘Shuimi’ | homozygous difficult-flowering type | 87.33% | 86.67% |
| 30 | ‘Yuhebao’ | homozygous difficult-flowering type | 61.33% | 0.00% |
| 31 | ‘Jianye’ | homozygous difficult-flowering type | 95.33% | 0.00% |
| 32 | ‘Jizuili’ | homozygous difficult-flowering type | 99.33% | 0.00% |
| 33 | ‘Baitangying’ | homozygous difficult-flowering type | 93.33% | 26.00% |
| 34 | ‘Baili’ | homozygous difficult-flowering type | 79.33% | 16.67% |
| 35 | ‘Miaozhongnuo’ | homozygous difficult-flowering type | 82.67% | 27.33% |
| 36 | ‘Dajingzhong’ | homozygous difficult-flowering type | 86.67% | 0.00% |
| 37 | ‘Xuehuaizi’ | homozygous difficult-flowering type | 88.67% | 0.00% |
| 38 | ‘Shakengzhong’ | homozygous difficult-flowering type | 63.33% | 40.67% |
| 39 | ‘Liuyuexue’ | homozygous difficult-flowering type | 91.33% | 0.00% |
| 40 | ‘Guanyinlv’ | homozygous difficult-flowering type | 85.33% | 8.00% |
| 41 | ‘Seedling tree-2’ | homozygous difficult-flowering type | 83.33% | 12.67% |
| 42 | ‘Zili’ | homozygous difficult-flowering type | 96.67% | 0.00% |
| 43 | ‘Ziniangxi’ | homozygous difficult-flowering type | 79.33% | 0.00% |
| 44 | ‘Shangshuhuai’ | homozygous difficult-flowering type | 86.00% | 24.00% |
| 45 | ‘Seedling tree-3’ | homozygous difficult-flowering type | 51.33% | 0.00% |
| 46 | ‘Kaleka’ | homozygous difficult-flowering type | 73.33% | 85.33% |
| 47 | ‘Qingpitian’ | homozygous difficult-flowering type | 76.67% | 0.00% |
| 48 | ‘Jinyinbao’ | homozygous difficult-flowering type | 93.33% | 0.00% |
| 49 | ‘Hexiacuan’ | homozygous difficult-flowering type | 85.33% | 0.00% |
| 50 | ‘Yulindingxiang’ | homozygous difficult-flowering type | 100.00% | 68.00% |
| 51 | ‘Lingshanxiangli’ | homozygous difficult-flowering type | 98.67% | 0.00% |
| 52 | ‘Seedling tree-4’ | homozygous difficult-flowering type | 97.33% | 0.00% |
| 53 | ‘Nanfeihuaizhi’ | homozygous difficult-flowering type | 99.33% | 0.00% |
| 54 | ‘Seedling tree-4’ | homozygous difficult-flowering type | 91.33% | 0.00% |
| 55 | ‘Seedling tree-5’ | homozygous difficult-flowering type | 79.33% | 0.00% |
| 56 | ‘Seedling tree-6’ | homozygous difficult-flowering type | 97.33% | 0.00% |
| 57 | ‘Seedling tree-7’ | homozygous difficult-flowering type | 92.67% | 0.00% |
| 58 | ‘Seedling tree-8’ | homozygous difficult-flowering type | 82.00% | 44.67% |
| 59 | ‘Seedling tree-9’ | homozygous difficult-flowering type | 99.33% | 0.00% |
| 60 | ‘Seedling tree-10’ | homozygous difficult-flowering type | 88.00% | 30.00% |
| 61 | ‘Seedling tree-11’ | homozygous difficult-flowering type | 81.33% | 80.00% |
| 62 | ‘Wuheli’ | Homozygous difficult-flowering type | 95.33% | 50.67% |
| 63 | ‘Huangdijiu’ | heterozygous type | 100.00% | 100.00% |
| 64 | ‘Wuyejiu’ | heterozygous type | 98.67% | 100.00% |
| 65 | ‘Fenghua’ | heterozygous type | 96.00% | 94.00% |
| 66 | ‘Kulin’ | heterozygous type | 99.33% | 100.00% |
| 67 | ‘Guilin’ | heterozygous type | 100.00% | 60.00% |
| 68 | ‘Wuye’ | heterozygous type | 90.00% | 69.33% |
| 69 | ‘Heiye’ | heterozygous type | 100.00% | 88.00% |
| 70 | ‘Jinzhong’ | heterozygous type | 100.00% | 100.00% |
| 71 | ‘Ruanzhizaohong’ | heterozygous type | 100.00% | 100.00% |
| 72 | ‘Xiafanzhi’ | heterozygous type | 97.33% | 100.00% |
| 73 | ‘Caikengrouwan’ | heterozygous type | 98.00% | 10.00% |
| 74 | ‘Shiyueli’ | heterozygous type | 96.00% | 82.67% |
| 75 | ‘Shuilin’ | heterozygous type | 91.33% | 59.33% |
| 76 | ‘Dahongpao’ | heterozygous type | 100.00% | 100.00% |
| 77 | ‘Zhumuru’ | heterozygous type | 100.00% | 100.00% |
| 78 | ‘Chenzi’ | heterozygous type | 100.00% | 100.00% |
| 79 | ‘Nanxizaosheng’ | heterozygous type | 100.00% | 100.00% |
| 80 | ‘Songjiaxiang’ | heterozygous type | 99.33% | 97.33% |
| 81 | ‘Seedling tree-1’ | heterozygous type | 98.67% | 94.67% |
| 82 | ‘Fenhongguiwei’ | heterozygous type | 97.33% | 99.33% |
| 83 | ‘Zuangyuanhong’ | heterozygous type | 98.00% | 100.00% |
| 84 | ‘Baibozaohong’ | heterozygous type | 93.33% | 91.33% |
| 85 | ‘Xiaojinzhong’ | heterozygous type | 100.00% | 93.33% |
| 86 | ‘Lingfengnuo’ | heterozygous type | 96.00% | 97.33% |
| 87 | ‘Shuidongheye’ | heterozygous type | 99.33% | 100.00% |
| 88 | ‘Guizaoli’ | heterozygous type | 100.00% | 100.00% |
