# Supplementary material for: Development of molecular markers based on the promoter difference of LcFT1 to discriminate easy- and difficult-flowering litchi germplasm resources and its application in crossbreeding
Source: BMC Plant Biol. 2021 Nov 16;21:539. doi: 10.1186/s12870-021-03309-7 (PMC8594225; doi:10.1186/s12870-021-03309-7)
Supplement: Supplementary file 5 — Additional file 5: Table S2. Primers used in this study. [file 12870_2021_3309_MOESM5_ESM.doc]

**Supplemental Table 2.** Primers used in this study

| Primer pairs used for cloning, molecular markers, expression analyses and construction of expression vectors | |
| --- | --- |
| *LcFT1*_F1 | TGAAAAGTATGAATCACAAAAAAGT |
| *LcFT1*_R1 | CAATAGCAAGAAGAGTAGAAGAAGA |
| E*LcFT1*_F | TTAATTAACATTAATTAATTAATTAATTAATTATAATTTGACACG |
| E*LcFT1*_R | GAGACACAATCTACAATGCACAGGAGTATGT |
| D*LcFT1*_F | TGATCAAAGAACATATAATATTTGGGCG |
| D*LcFT1*_R | TAAATAAAAATACTCATTATATATGAGC |
| CE*LcFT1*_F | TTGTTGGCGTTGTCGGTTCTAAT |
| CE*LcFT1*_R | AGCTAAGCAGCCACAAACTCAAT |
| *LcFT1*_F2 | GGTGGAAGGAGACGATAAAGTCC |
| *LcFT1*_F2 | TAGTACCGGGGATAATGGAGGAG |
| *Lcactin*_F | ACCGTATGAGCAAGGAAATCACTG |
| *Lcactin*_R | TCGTCGTACTCACCCTTTGAAATC |
| *LcFT1*_F3 | GCAGGTCGACTCTAGATGAAAAGTATGAATCACAAAAA |
| *LcFT1*_R3 | AGGGACTGACCACCCGGGGATCCGAAATGCACAACAATAG |
